# Supplementary material for: Response to Fingolimod in Multiple Sclerosis Patients Is Associated with a Differential Transcriptomic Regulation
Source: Int J Mol Sci. 2024 Jan 23;25(3):1372. doi: 10.3390/ijms25031372 (PMC10855583; doi:10.3390/ijms25031372)
Supplement: Supplementary file 1 [file ijms-25-01372-s001.zip › TABLE S1.pdf]

Table S1. Baseline demographic characteristics of each healthy donor (HD)

Healthy donor	Sex	Age
HD_1	Male	60
HD_2	Female	35
HD_3	Female	32
HD_4	Female	43
HD_5	Female	30
HD_6	Male	58
HD_7	Male	47
HD_8	Female	42
HD_9	Female	51
HD_10	Female	35
