# Supplementary material for: Response to Fingolimod in Multiple Sclerosis Patients Is Associated with a Differential Transcriptomic Regulation
Source: Int J Mol Sci. 2024 Jan 23;25(3):1372. doi: 10.3390/ijms25031372 (PMC10855583; doi:10.3390/ijms25031372)
Supplement: Supplementary file 1 [file ijms-25-01372-s001.zip › TABLE S2.pdf]

Table S2. Baseline demographic and clinical characteristics of each multiple sclerosis patient

MS patient	Sex	Age	Disease duration (years)[1]	Time since DMT onset (years)	Nº of previous DMTs	Immediatley previous DMT	EDSS
Responder_1	Female	25	4.6	0.6	1	Interferon beta	0
Responder_2	Female	42	14.8	5.6	2	Natalizumab	5.5
Responder_3	Male	49	3.1	2.4	1	Dimethyl fumarate	0
Responder_4	Male	56	6.8	2.2	1	Natalizumab	3.5
Responder_5	Male	28	2.3	2.0	1	Natalizumab	1.5
NonResponder_1	Female	32	14.0	13.0	3	Natalizumab	2.5
NonResponder_2	Female	36	0.8	0.7	1	Natalizumab	0
NonResponder_3	Male	37	10.0	0.7	1	Interferon beta	2
NonResponder_4	Female	42	4.0	3.9	2	Natalizumab	1
NonResponder_5	Female	20	3.2	2.8	1	Dimethyl fumarate	0

[1]Time since the first symptoms of multiple sclerosis (MS)

DMT, disease-modifying treatment; EDSS, expanded disability status scale
